# The Science and the Art of Surgery

**Published:** 1854-11

**Authors:** 


**THE SCIENCE AND ART OF SURGERY.** By John Erickson, Professor of Surgery in the University College, and Surgeon to the University College Hospital, London, 1854.

This department of medical science has been advancing with rapid strides in the few past years, and its literature keeps pace with the other improvements. Among the recent valuable contributions the above work stands prominently forward. It is edited by John H. Brinton, M. D., of Philadelphia, who has contributed largely to its value. Few works are written in a style so clear and perspicuous, and the three hundred and upward illustrations render the text still better understood. The pathological changes in the blood in inflammation, the condition of its white and red particles under the different stages and changes, are well described and represented.—The colorless corpuscle adhering to the walls of the blood vessel, the continued movement of the red globules and their accumulation and arrest, are clearly enough described in the text, but are further shown by the illustrations in connexion.

The chapter on operations should be perused by all whether by those who make pretensions to the use of the knife or the *tyro* whose ambition looks in that direction. The author gives a wise and judicious caution in the use of chloroform, and the American editor concludes his remarks upon it by a note at the close of which he says: "The numerous fatal consequences, however, which have attended and are daily resulting from the employment of chloroform in Europe have led to the almost entire abandonment of its use in this country." He gives a preference to well washed ether as an anæsthetic and maintains that it is less dangerous. The work is a valuable contribution to medical literature and should be found among the other standard works in the physicians' library. The *ear* and the *eye* have no part in this work, from the fact that they have been made the subjects of so many special treatises.

This work, one among the best extant, contains 902 pages, and is published by Blanchard & Lea, Philadelphia, 1854.

